# Detection and Quantification of *Rhizoctonia solani* and *Rhizoctonia solani* AG1-IB Causing the Bottom Rot of Lettuce in Tissues and Soils by Multiplex qPCR

**DOI:** 10.3390/plants10010057

**Published:** 2020-12-29

**Authors:** Thérèse Wallon, Andréanne Sauvageau, Hervé Van der Heyden

**Affiliations:** Department of Phytopathology and Biosurveillance, Phytodata, 291 rue de la Coopérative, Sherrington, QC J0L 2N0, Canada; Therese.Wallon@USherbrooke.ca (T.W.); asauvageau@phytodata.ca (A.S.)

**Keywords:** bottom rot of lettuce, *Rhizoctonia solani*, AG 1-IB, AG-BI, multiplex qPCR, plant detection, soil quantification

## Abstract

In the muck soil region of southwestern Quebec, vegetable growers are threatened by several soilborne diseases, particularly the bottom rot of lettuce caused by the fungus *Rhizoctonia solani*. The particularly warm temperature of the few last seasons was marked by an increase in disease severity, and the associated yield losses were significant for Quebec lettuce growers. In the absence of registered fungicides and resistant cultivars, the management of *Rhizoctonia solani*-induced diseases in lettuce is based on good agricultural practices, which require detailed knowledge of the pathogen. In this study, *Rhizoctonia solani* fungal strains were isolated from infected field-grown lettuce plants presenting bottom rot symptoms to determine the anastomotic groups (AGs) of these isolates by internal transcribed spacer region (ITS) sequencing. *Rhizoctonia solani* AG 1-IB was identified as the main anastomotic group causing bottom rot lettuce in field-grown lettuce in organic soils in the Montérégie region. Two specific and sensitive quantitative PCR assays were then developed for *R. solani* AG1-IB and *R. solani*. The AG 1-IB qPCR assay amplified all strains of *R. solani* AG 1-IB tested, and no PCR product was obtained for any non-target strains. The *R. solani* qPCR assay amplified all strains of *R. solani* and did not amplify non-target strains, except for two strains of binucleate *Rhizoctonia* AG-E. In artificially inoculated soils, the sensitivity of both qPCR assays was set to 1 μg of sclerotia g^−1^ of dry soil. In the growth chamber experiment, a minimum concentration between 14 and 42 μg sclerotia g^−1^ of dry soil was required to induce the development of symptoms on the lettuce. Indeed, the AG 1-IB qPCR assay was sensitive enough to detect the lowest soil concentration of AG1-IB capable of inducing symptoms in head lettuce. In addition, the qPCR assays successfully detected *R. solani* and *R. solani* AG 1-IB from infected plant tissue samples and soil samples from lettuce fields. The qPCR assays developed in this study will be useful tools in lettuce bottom rot management.

## 1. Introduction

In southwestern Québec (Canada), lettuce is an important crop with an annual production of about 3500 metric tons, representing 62% of Canada’s production [1]. In this area, lettuce is mainly grown in muck soils from late April to early October, predominantly through the use of transplants. This production is threatened by several soilborne diseases. In the spring, pythium stunt caused by *Pythium tracheiphilum* is the most important disease [2], while bottom rot caused by *Rhizoctonia solani* is predominant throughout the summer*. Rhizoctonia solani* (Kühn) is a ubiquitous soilborne plant pathogen capable of infecting the seedlings, roots, leaves, and stems of a multitude of vegetable plants, causing a variety of diseases [3]. The first symptoms often occur as golden-brown lesions on leaf ribs and old leaves in contact with the soil, the main reservoir of the *R. solani* inoculum. Under conductive conditions, mycelial growth resumes from sclerotium, the over-seasoning structures, and the mycelium spreads until it meets the host plant tissue [4]. When temperatures and humidity are high, these lesions can quickly develop into wet rot, starting on the leaf ribs and moving toward the plant head, causing the greatest damage close to harvest. In eastern Canada, the number of high heat episodes tends to increase during the months of July and August, increasing bottom-rot incidence and severity. In southwestern Québec, no synthetic fungicides are registered against *R. solani*—only *Bacilius subtilis*-based biofungicides are currently available to growers. Moreover, there are no resistant lettuce cultivars available. Hence, the principal management method for *R. solani* in lettuce production is crop rotation, which in southwestern Québec mainly includes onions and carrots. Worldwide, the production areas in the United States, Brazil, and Germany also experienced outbreaks of basal rot caused by different anastomosis groups (AGs) of *Rhizoctonia solani* [5,6,7,8].

*The Rhizoctonia* complex consists of binucleate *Rhizoctonia* and multinucleate *Rhizoctonia solani*, each subdivided into several groups based on their hyphal anastomosis reactions. Binucleate *Rhizoctonia* are divided into 22 AGs (AG-A to AG-W), but, although some of them are associated with root rot in strawberry, potato, and soybean, the pathogenicity of these AGs varies greatly from isolate to isolate and many have been reported to have a neutral or beneficial relationship with the plant [9,10,11]. *Rhizoctonia solani* Kühn (teleomorph *Thanatephorus cucumeris*) is divided into 14 distinct anastomotic groups, AG-1 to AG-13, and AG-BI, which are also divided into subgroups (i.e., AG 1-IA, IB, IC, ID, IE, IF) [12,13]. Anastomosis groups can be distinguished by a distinct host range and virulence levels in crops, while the internal transcribed spacer region (ITS) has been identified as a useful barcode for the identification of *R. solani* to their respective AGs [12,14,15]. The anastomosis groups isolated thus far from greenhouses or field-grown lettuce worldwide include AG 1, AG 1-IB, AG 1-IC, AG 2, AG 2-1, AG 2-1Nt, AG 2-2, AG 3, AG4, AG4-HGI, AG 5, AG10, and AG-BI, as well as some unidentified AGs [5,7,8,15,16,17,18]. In the field AG1-IB, AG 1-IC, AG 2-1, AG 2-2, AG 4, and AG 5 groups were isolated in the muck soil of Ohio (USA); AG 1-IB, AG 1-IC, and AG 2-1 were isolated in Germany; and AG 1-IA and AG 1-IB were isolated in Brazil [5,7,8,19]. These studies showed that AG 1-IB is the most prevalent AG associated with bottom rot in field-grown lettuce. Hence, precise identification of the predominant *R. solani* AG may help implement new management strategies against bottom rot in lettuce crops [20].

Integrated pest management (IPM) relies on a combination of different approaches (e.g., cultivar selection, crop rotation, and biocontrol agents) to keep the populations of pathogens below the economic damage threshold [21,22,23]. For soilborne diseases, several promising IPM strategies are based on the quantification of soilborne pathogen inoculum [24,25]. In the specific case of *Rhizoctonia*-induced diseases, quantification of the soil inoculum was found to be useful in different contexts. For example, it was used to assess rice cultivar susceptibility to *R. solani* AG 1-IA [26] to assess the impact of different crop rotations on the inoculum levels of a specific AG and its main host plant [27,28], and to map the inoculum level at the field scale to make site-specific fungicide applications to limit the local transmission of the pathogen [29]. A positive correlation between *R. solani* soilborne inoculum and disease incidence or severity was found for several *R. solani* AGs and their host plants under controlled experiments [19,30], but fewer studies confirmed this relationship in field experiments [31]. *Rhizoctonia solani* inoculum can be assessed by baiting methods or with the use of molecular detection assays [27,32,33,34]. Since the molecular method has the advantage of being rapid and able to be designed specifically for a given *R. solani* AG, unlike baiting methods, it was used to quantify the *R. solani* soilborne inoculum in various soil types [32,33]. The implementation of such soilborne inoculum quantitation protocols enables the development of damage thresholds, allowing us to make better decisions regarding field selection [2,35]. Hence, this study aims to (i) identify the predominant *R. solani* AG responsible for lettuce basal rot in the muck soils of southwestern Québec; (ii) develop qPCR assays specific to *R. solani* and *R. solani* AG1-IB in order to detect and quantify them in soil and lettuce tissue samples; (iii) develop a molecular detection threshold for *R. solani* inoculum in muck soil; and (iv) investigate the relationship between the AG1-IB soil inoculum concentration and disease development under controlled conditions to validate the biological significance of the molecular detection threshold found.

## 2. Results

### 2.1. Characterization of Rhizoctonia solani Isolates

**Figure 1:**
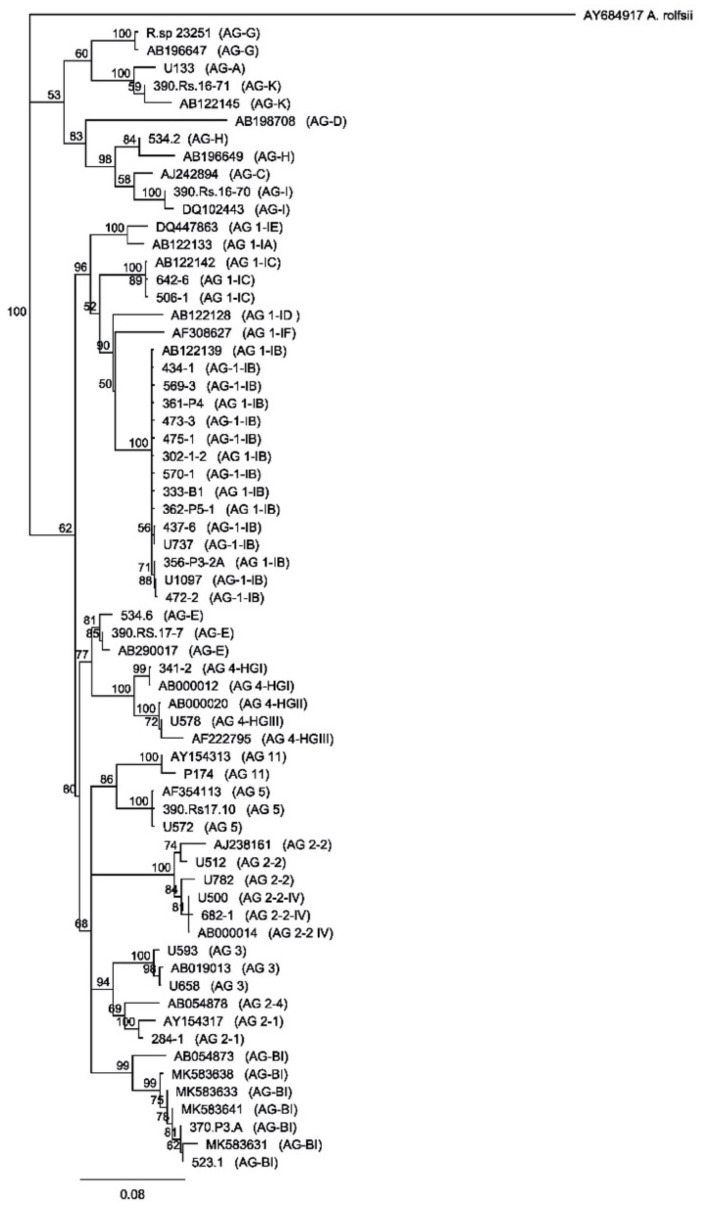
Neighbor-joining consensus tree based on the alignment of 65 ITS sequences of *Rhizoctonia solani* and binucleate *Rhizoctonia*. Twenty-eight sequences were retrieved from Genbank, 17 were from lettuce isolates, 20 were isolated from other crops, and Athelia rolfsii (AY684917) was used as an outgroup. Bootstraps values (1000 replicates) are indicated only for branches with a value higher than 50.

Sequencing of the internal transcribed spacer region (ITS) conducted on 60 *R. solani* isolates collected from infected lettuce showed that 95% of the isolates belonged to AG 1-IB, 3.3% to AG-BI, and 1.7% to AG 1-1C (Figure S1). The closest sequences to our AG-BI isolates came from pathogenic AG-BI strains isolated from lettuce in Belgian greenhouses [16] (Supplementary Figure S2). Isolates AG-BI 370-P3-A and 523-1 (Table 1) showed 100% and 99.8% identity with AG-BI MK583630, respectively. The *R. solani* AG-BI strains were isolated from romaine lettuce samples collected from two different fields on the same farm, while *R. solani* AG1-IC was isolated from iceberg lettuce. The phylogeny of *R. solani* ITS sequences obtained from the isolates collected from infected lettuce leaves allowed the identification of different AGs (Figure 1). All the sequences obtained in this study were deposited in the GenBank database (accession MT177217-MT177269).

### 2.2. Development of the Rhizoctonia solani- and Rhizoctonia solani AG1-IB-Specific qPCR Assays

#### 2.2.1. Primer and Probe Design

The alignment of 87 *R. solani* and Binucleate *Rhizoctonia* ITS sequences obtained in this study and from the NCBI database was used to design the qPCR primers and probe sets (Supplementary Figure S3). Primers and probes used in this study are presented in Table 2. The previously described primers of GMRS3-R and a modified version of the GMRS4 primer (GRSM4_m_), combined with the newly developed probe (GRMP) (Supplementary Figure S4), amplified a PCR product ranging from 87 to 109 bp, depending on the AG (Supplementary Figures S4 and S8) [36]. For *R. solani* AG 1-IB, the ITS1 region specifically allowed us to differentiate between the members of the AG 1 subgroup, including AG 1-IA, -IB, -IC, -1D, -IE, and -IF (Supplementary Figure S5). The primers AG 1-IB-F3 and AG 1-IB-R and the probe AG 1-IB-P were designed on the 5′ end of the ITS1 region (Supplementary Figure S6) and amplified a 90 bp PCR product (Supplementary Figure S7). For the AG 1-IB assay, the specificity was largely conferred by reverse and forward primers.

#### 2.2.2. Primer and Probe Specificity

The *R. solani* assay amplified DNA from 11 different *R. solani* AGs, including AG 1-IB, 1-IC, AG 2-1, 2-2, 2-2-IV, AG 3, AG 4-HGI, AG 4-HGII, AG 5, AG 11, and AG-BI, with Cq values ranging from 21.58 to 30.45. No amplification was recorded for Binucleate *Rhizoctonia* AG-A, AG-G, AG-H, AG-I, AG-K, or for the other tested fungi. The only unexpected amplification came from the Binucleate *Rhizoctonia* AG-E group, for which isolates 390.Rs17-7 and 534-6 were positive, with a Cq value of 25.69 and 27.24, respectively (Table 1). The *R. solani* AG 1-IB assay only amplified DNA from the 14 *R. solani* AG 1-IB isolates tested, with Cq value ranging from 21.59 to 25.20 (Table 1). No amplification was recorded for the other 10 AGs tested, for the six Binucleate *Rhizoctonia*, or for the other fungi.

#### 2.2.3. Primer and Probe Efficiency and Sensitivity

**Figure 2:**
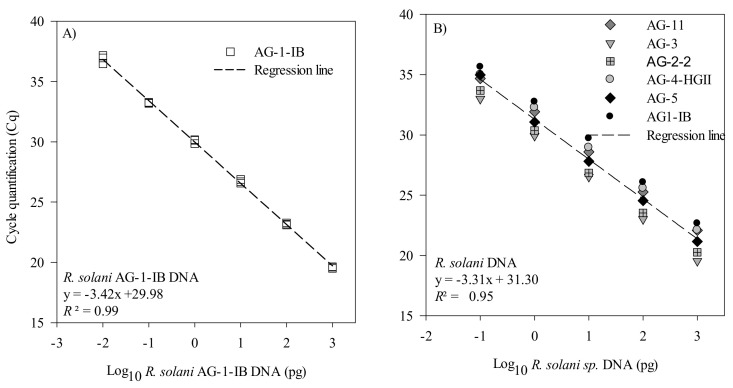
Standard curves and regression of *Rhizoctonia solani* AG 1-IB and *R. solani* dilution series measured by qPCR. The cycle quantification threshold (Cq) is plotted against the log_10_ of (**A**) the picogram of *R. solani* AG 1-IB DNA belonging to several isolates. The results are from three independent experiments and (**B**) the picogram of *R. solani* DNA from isolates belonging to the AG1-IB, AG2-2, AG3, AG4-HGII, AG5, and AG11 groups. Cq values for these experiments are provided in Supplementary Tables S1 and S2.

The efficiency and sensitivity of the *R. solani* and *R. solani* AG 1-IB qPCR assays were first evaluated using a 10-fold serial dilution of a gDNA solution. For the AG 1-IB assay, the standard curve showed a linear dynamic range of amplification over six orders of magnitude with a slope of −3.42, corresponding to an efficiency of 95.6% (Figure 2A). For the *R. solani* assay, the standard curve showed a linear dynamic range of amplification over five orders of magnitude with a slope of −3.31, corresponding to an efficiency of 100.5% (Figure 2B). The limit of detection was 0.01 pg of DNA and 0.1 pg of DNA for the AG 1-IB and the *R. solani* assays, respectively.

**Figure 3:**
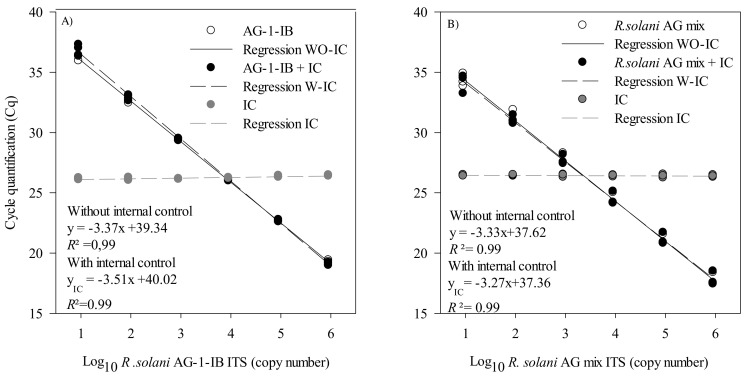
Standard curves and regression of *Rhizoctonia solani* AG 1-IB and *R. solani* dilution series measured by qPCR. The cycle quantification threshold (Cq) is plotted against the log_10_ of (**A**) the *R. solani* AG1-IB ITS copy number with and without the EIPC100 internal control (IC) and (**B**) the number of *R. solani* AG-mix ITS copies with and without the EIPC100 IC. The results are from three independent experiments. These experiments were conducted using three biological replicates and two technical replicates for each biological replicate. Cq values for these experiments are provided in Supplementary Tables S3 and S4.

Both assays were multiplexed with an internal control to detect false negatives, which sometimes occur due to the presence of soil or plant inhibitors in environmental DNA extracts. For the *R. solani* AG1-IB assay, the slope of the regressions was slightly greater with the IC (−3.51) compared to the regression obtained without the IC (−3.37) (Figure 3A). For the *R. solani* assay, the slopes of the regressions were similar both with (−3.33) and without the IC (−3.27) (Figure 3B). The addition of the IC in a multiplex reaction did not interfere with the initial *R. solani* or *R. solani* AG 1-IB quantification or detection limit.

#### 2.2.4. Validation in Artificially Inoculated Soil with *R. solani* AG 1-IB and *R. solani* Sclerotia

**Figure 4:**
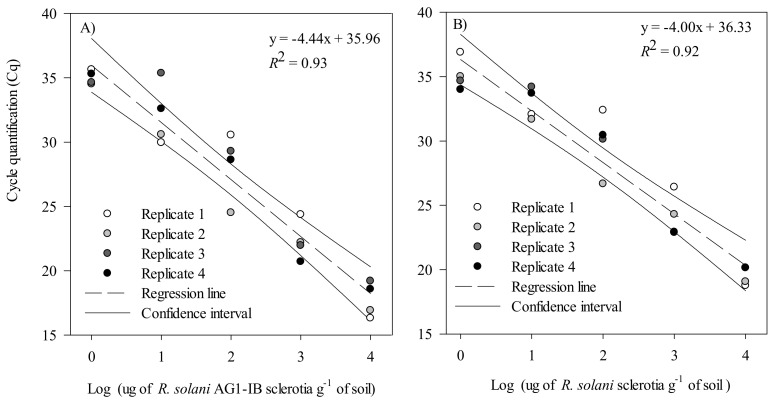
Linear regression of the mean cycle threshold (Cq) value plotted against the log10 of sclerotia (μg sclerotia g^−1^ of dry soil) of (**A**) *Rhizoctonia solani* AG1-IB and (**B**) *R. solani*, obtained from the artificial inoculation of sterilized muck soils. These experiments were conducted using four biological replicates and two technical replicates for each biological replicate. Cq values for these experiments are provided in Supplementary Table S5.

The validation conducted with artificially inoculated soils showed a negative linear relationship between the log10 of the sclerotia concentration in the soil (µg g^−1^ of soil) and the qPCR cycle threshold for both *R. solani* AG 1-IB and *R. solani* (Figure 4A,B). For the *R. solani* AG 1-IB assay, the slope of the linear regression was −4.44 (*R*^2^ = 0.92), and −4.00 (*R*^2^ = 0.93) for the *R. solani* assay. Moreover, for both assays, the ANOVA conducted with four independent replicates followed by an LSD test showed that all concentrations were significantly different from each other (*p* < 0.001). Hence, combining the soil conditioning method, DNA extraction protocol, and qPCR conditions provided a limit of detection of 1 μg sclerotia g^−1^ of dry soil.

### 2.3. Investigation of the Relationship between R. solani AG 1-IB Soil Inoculum and Disease Development

**Figure 5:**
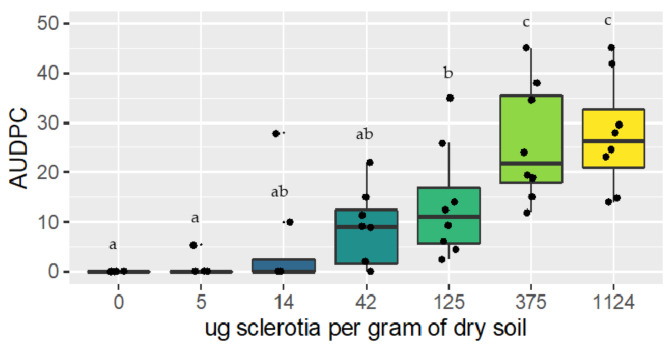
Results of the artificial inoculation trials performed under controlled conditions for *R. solani* AG 1-IB. Each boxplot shows the distribution of the combined results of the two experiments, with different letters indicating a significant difference between treatments according to an LSD test.

For both trials, the area under the disease progress curve (AUDPC) increased as the soil inoculum concentration increased. Lettuce plants grown in non-inoculated soil did not show any basal rot symptom, except for one plant in trial two that may have been contaminated by a neighboring plant. The ANOVA conducted in both trials suggested that there was a significant effect of soilborne inoculum on disease development (*p* < 0.001) (Figure 5). The results of this experiment suggest that concentrations above 125 μg of sclerotia per gram of soil produce more symptoms than concentrations of 0 and 4 μg of sclerotia per gram of soil, while concentrations of 14 and 42 μg of sclerotia per gram of soil belong to both groups (Figure 5).

### 2.4. Validation Using Naturally Infested Plant and Soil Samples

#### 2.4.1. *Rhizoctonia solani* and *Rhizoctonia solani* AG1-IB Detection in Plant Tissue

To validate the use of qPCR assays on the infected lettuce collected in the field, 30 samples of plant material were selected. For each of the tested samples, *R. solani* was previously isolated and identified in the anastomotic group by ITS sequencing, as described above (Table 3). All plant tissue samples from which *R. solani* AG 1-IB was isolated were positive for both *R. solani* and *R. solani* AG 1-IB using the qPCR assays developed in this study. The three samples in which *R. solani* AG1-1C or AG-BI were isolated were positive in the *R. solani* qPCR assay but negative for the *R. solani* AG 1-IB assay.

#### 2.4.2. *Rhizoctonia solani* and *Rhizoctonia solani* AG1-IB Soil Distribution

**Figure 6:**
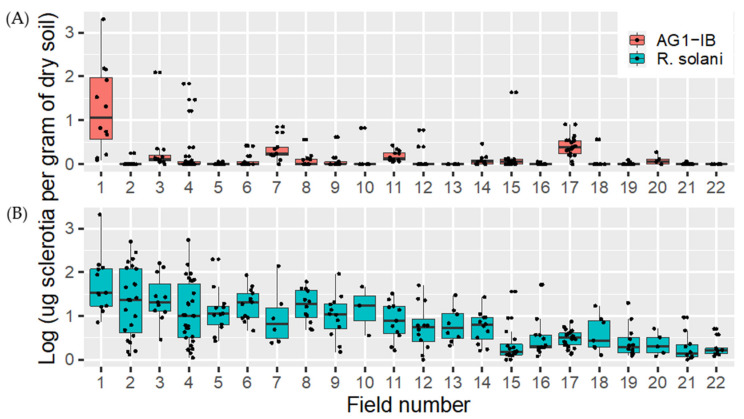
Box plot diagram showing the amount of (**A**) *R. solani* AG-1-IB log10 μg sclerotia per gram of dry soil and (**B**) *Rhizoctonia solani* log μg sclerotia per gram of dry soil detected by qPCR methods in commercial fields sampled in spring 2017 and 2018 prior to lettuce planting. The fields are placed in ascending order of their mean value for *R. solani*.

In 2017 and 2018, a total of 284 soil samples were collected in 22 fields. These soil samples were processed and tested using the method described in this study for both *R. solani* and *R. solani* AG 1-IB. Globally, 98.5% of the samples tested were positive for *R. solani,* with concentrations ranging from 0 to 2082.9 μg of sclerotia per gram of dry soil, while 43.3% were positive for *R. solani* AG 1-IB, with concentrations ranging from 0 to 2010.5 μg of sclerotia per gram of dry soil (Figure 6). As expected, the average concentration of *R. solani* AG-1-IB was lower than the concentration measured for *R. solani*, suggesting that most of the DNA detected in the samples tested belong to other anastomotic groups.

## 3. Discussion

In Canada and elsewhere, the main method of controlling lettuce basal rot is based on crop rotation. In this context, it is essential to keep the inoculum concentration as low as possible. Thus, accurate knowledge of the anastomosis groups present and reliable quantification can allow better monitoring and contribute to improved and more efficient management.

The results obtained in this study suggest a high prevalence of *R. solani* AG1-IB causing bottom rot in Quebec lettuce. This is consistent with the findings from other regions of the world where *R. solani* AG 1-IB is highly prevalent and virulent in field lettuce [5,7,19]. *Rhizoctonia solani* AG 1-IC and AG-BI were also found in Quebec lettuce. The presence of *R. solani* AG 1-IC has also been reported in the United States and Germany [5,7]. This is the second study reporting the isolation of *R. solani* AG-BI from a diseased lettuce plant in the field.

Indeed, AG-BI is mostly found in soil and has long been considered non-pathogenic or weakly pathogenic when tested in a pathogenicity assay, but it was recently isolated from symptomatic plants on lettuce grown in greenhouse soil [12,16]. Studies have reported higher virulence and greater symptoms of AG 1-IB in lettuce compared to AG 1-1C and AG-BI [5,16]. *Rhizoctonia solani* AG 1-IC has also been shown to be pathogenic on lettuce [37]. Although not all *R. solani* AG1-IB isolates showed the same degree of virulence, several strains were able to infect broccoli, spinach, and radish [7]. All of these crops are grown in the Québec muck soil region and are likely to be part of the crop rotation plan with lettuce. Such crops could, therefore, constitute a reservoir for AG1-IB and help maintain a certain level of inoculum in the soil.

Following identification of the predominant AG associated with basal rot symptoms in Québec lettuce production, a molecular assay was developed for the detection of *R. solani* AG1-IB from plant tissues and soil samples. The ITS region has already been used for the detection of *R. solani* and was proven to be an appropriate barcode to resolve the AG-1 subgroup [38]. SCAR primers were previously developed for AG1-IB but were not adapted for soil samples and could not be quantitative [38]. The ITS region showed great diversity among its *R. solani* AGs, which allowed the successful design of a specific primer and probe set. The specificity of the assay was shown by testing several *R. solani* isolates representing a wide range of AGs from multinucleate and binucleate *Rhizoctonia*. Although our results suggest that AG1-IB is primarily responsible for lettuce basal rot, several other AGs can also infect lettuce in different growing regions. It was, therefore, important to provide a tool to capture as many AGs as possible while excluding binucleate *Rhizoctonia* (AGs), as these AGs are often non-pathogenic or reported to have a neutral or beneficial relationship with the plant [11]. All multinucleate strains were positive with the *R. solani* assay, but two AG-E strains of Binucleate *Rhizoctonia* were also positive (Table 2). Nevertheless, AG-E has a wide host range and has been reported on several crops, including lettuce, so it may be useful to include it when detecting *R. solani* in the environment [3]. Moreover, this assay represents an improvement of the previously described SYBR green assay, as it allows absolute quantification of the *R. solani* inoculum in plant and soil material [36,39].

Obtaining an accurate diagnosis is essential for adjusting integrated management strategies. Although it is relatively simple to identify *R. solani* on a culture medium, determining the anastomosis group using such techniques remains relatively complex. For the field samples collected in this study, the results obtained by isolation on a culture medium combined with ITS sequencing were consistent with the detection obtained by qPCR conducted with lettuce tissues. In general, the qPCR-based assay allowed a faster diagnosis than microbiological methods. Thus, when used in pairs, the qPCR assays proposed in this study confirm the presence of *R. solani* on rot lesions and determine whether this is the predominant AG 1-IB or not. The use of the *R. solani* marker alone may lead to false-positive results but since AG-BI, AG-1-IC, and other AGs can induce symptoms, it remains important to use this marker. When the sample is positive for *R. solani* and negative for AG 1-IB, an ITS1-GRSM4 PCR followed by sequencing can be performed to determine the AG present in the sample, thereby avoiding the use of the microbiological isolation method [40].

In addition to high specificity, the tests that were developed in this project had a high sensitivity. The protocol developed in this study allows the detection of 1 μg of sclerotia per gram of dry soil for *R. solani* and *R. solani* AG 1-IB. A SCAR marker was previously developed for AG1-IB, which allowed detection in soils, but the detection limit was 0.01 g sclerotia per gram of soil. The method proposed here, therefore, represents an improvement over the latter, since it offers sensitivity more than 5 logs higher than the previous method. The detection limit obtained in this study can be compared to other similar studies where the detection limit was around 0.1 μg sclerotia per gram of soil for, e.g., AG2-1, and 500 μg sclerotia per gram of soil for AG3 [32,33]. Several research groups have developed molecular assays to specifically detect anastomotic groups in different soil types, and all agree that the sensitivity of a molecular assay evaluated with field samples depends directly on the type of molecular technique used (PCR vs. qPCR) but also on the sampling techniques, the pre-treatments performed on the field sample before DNA extraction, and the DNA extraction technique itself [32,33]. Considering that *Rhizoctonia solani* often has an uneven distribution pattern in the field [32,41] and that *Rhizoctonia solani* is a sclerotia-producing fungus, this fungus is particularly difficult to reproduce with respect to DNA extraction without proper sample homogenization [24,42]. To illustrate the importance of homogenization and conditioning techniques prior to extraction, it is interesting to note that we obtained a detection threshold identical to that in a study by Budge et al. [33] using a DNA extraction of 0.2 g versus 4 g. In our protocol, we included a soil homogenization step with a mechanical method upstream of our extraction protocol. A total of 100 g of soil was crushed, which allowed us to crush the sclerotia and thus probably standardize the presence of the inoculum within the soil sample.

The results from growth chamber trials suggest that sclerotia concentrations between 5 and 14 μg per gram of soil can induce basal rot symptoms in lettuce, and for those above 42 μg sclerotia per gram of soil, the symptoms are significantly greater. The qPCR test developed in this study was effective in detecting up to 1 μg of sclerotia per gram of dry soil, which is well below the theoretical disease threshold found in the growth chamber trials. These results, therefore, suggest that the detection limit of the qPCR test developed in this project is sufficiently sensitive to be used for the development of risk indicators. The results obtained in this trial are supported by those obtained by Grosh and Kofoet [19], who demonstrated that there is a positive correlation between the concentration of artificially inoculated AG1-IB in the soil and the inhibition of lettuce growth.

Validation of the assays with environmental samples confirmed the ability of the assays to detect *R. solani* and AG1-IB in soils and reinforces the potential of these markers for biomonitoring and studying the epidemiology of this pathogen. It has been shown in the field that short rotations and soil temperatures influence the severity and frequency of field losses [31]. To develop an integrated risk management model, it will be necessary to correlate the development of symptoms in the field with the soil inoculum and also with other meteorological parameters since environmental factors are clearly as important as the presence of the inoculum itself.

## 4. Materials and Methods

### 4.1. Field Sampling

Plant and soil samples were collected from the muck soil of southwestern Québec, from 2015 to 2018. Iceberg and romaine lettuce plants showing basal rot symptoms were taken from 31 commercial lettuce fields belonging to 8 different growers. The collected plants were refrigerated at 4 °C prior to sample preparation and pathogen isolation. In 2017 and 2018, soil samples were also collected from a total of 22 fields belonging to 4 lettuce growers. Each soil sample was composed of 15 random soil subsamples collected within a 5 m × 5 m quadrant in the first 15 cm from the soil surface. Upon sampling, 100 g sub-samples were homogenized, poured into an aluminum plate, air-dried at room temperature for 48 h, and prepared for DNA extraction (see Section 4.3).

### 4.2. Rhizoctonia Solani Isolation and Culture Method

For each of the diseased lettuce plants collected, leaves showing bottom rot symptoms were washed under running water and cut into 10 mm discs. These discs were then placed in sterile water for 1 min, surface sterilized with a 1% sodium hypochlorite solution for 1 min, and rinsed again with sterile water for another minute. The sterilized leaf discs were placed on a water agar media (WA) and/or potato dextrose agar (PDA) amended with 0.1 g per liter novobiocin. Another 0.2 g of the remaining leaf discs was placed in a lysing matrix tube C (MP Biomedicals, Solon, OH, USA) and frozen until DNA extraction (see Section 4.3). Petri dishes containing the leaf discs were incubated at room temperature for 24 to 72 h, after which fresh mycelium taken from the edge of the growing colony was transferred onto a new Petri containing PDA without antibiotics. Once the *R. solani* isolates formed sclerotia, fresh mycelium was scratched from the surface of the Petri plate and placed in a sterile 2 mL tube with 100 mg of 500–750 µm bead glass and then frozen until DNA extraction.

### 4.3. Fungal, Plant, and Soil DNA Extraction

DNA from both mycelium and plant samples was extracted using the Fast DNA Spin Kit (MP Biomedicals, Solon, OH, USA) with a CLS-VF buffer, following the manufacturer’s instructions. For soil, the dried sub-samples were ground for 30 s using a mill grinder, and 0.2 g of the resulting powder was placed in a matrix tube D (MP Biomedicals, Solon, OH, USA). Total DNA was extracted using a Fast DNA Spin Kit for soil (MP Biomedicals, Solon, OH, USA) following the manufacturer’s instructions. Regardless of the sample type, the final DNA elution was performed using 100 uL of pre-heated (55 °C) provided elution buffer. The concentration and quality of each purified DNA sample were estimated using a Nano-Drop lite spectrophotometer (Thermo Scientific, Mississauga, ON, Canada). DNA extracts with a quantity above 10 ng/uL and A260/A280 ratio around 1.8 were kept.

### 4.4. Rhizoctonia Solani Isolate Characterization

The AG of each *R. solani* isolate was identified by sequencing the internal transcribed spacer region (ITS) [14]. The ITS region was amplified using the universal ITS1/ITS4 primers (Table 1) [42]. The amplification mix contained 500 nM of each primer, 1x of the Phusion High-Fidelity PCR Master Mix with HF Buffer (New England Biolab, Ipswich, MA, USA), 0.2 μg uL^−1^ of BSA, and 5 uL of the template DNA in a total reaction volume of 35 uL. The PCR cycling conditions were set to 98 °C for 2 min, 35 cycles at 98 °C for 10 s, 60 °C for 15 s, and 72 °C for 30 s, followed by a final extension step at 72 °C for 10 min. The PCR products were visualized by electrophoresis on an agarose gel and sent to the Centre de Recherche du CHUL/CHUQ at Laval University to be purified and sequenced using an ITS4 primer. Each DNA sequence was visualized and manually trimmed using Geneious V9.1.8 (Biomatters, Auckland, New Zealand). Sequences were aligned using the NCBI tool BLASTN to identify the sequence with the highest homology. To avoid misidentification, sequences were also aligned with a pool of *R. solani* ITS reference sequences using Geneious V9.1.8. A total of 60 *R. solani* isolates obtained from lettuce were sequenced in addition to other *R. solani* isolates collected from other crops (Figure 1).

### 4.5. Design of Rhizoctonia solani and Rhizoctonia solani AG1-IB qPCR Assays on ITS

A total of 87 *R. solani* and Binucleate *Rhizoctonia* ITS sequences obtained in this study or from the GenBank database were aligned using the MAFT alignment tool available in Geneious V9.1.8 [14,15,43]. Out of the *R. solani* alignment, a consensus nucleotide sequence unique to AG 1-IB was chosen for the primers and probe designed using Primer Express V3.0.1 (Life technologies, Carlsbad, CA, USA). A set of primers (AG 1-IB-F3 and AG 1-IB-R) and a probe (AG 1-IB-P) were obtained, producing a 90 pb PCR amplification product (Table 1). For *R. solani*, a probe (GRMP) was designed to be used with the previously described GMRS3-R PCR primer [38] and a slightly modified version of the GMRS4 reverse primer (Table 1) (Johanson, 1998). Potential secondary structures, such as hairpin, self-dimer, and hetero-dimer interactions, were checked using the oligoanalyzer tool from Integrated DNA Technologies (https://www.idtdna.com/pages/tools/oligoanalyzer). The specificity of the primers and probe set was first verified by a BLASTN analysis of each sequence in the NCBI database (https://blast.ncbi.nlm.nih.gov/Blast.cgi).

### 4.6. qPCR Conditions of Rhizoctonia solani and Rhizoctonia solani AG-1-IB Assays

For the *R. solani* simplex assay, the qPCR was conducted in a final volume of 25 uL containing 1X ECO master mix (Thermofisher, Mississauga, On, Canada), 500 nM each of the GRSM3 and GRSM4 primers, 250 nM of the GRMP probe, 0.2 μg uL^−1^ of BSA, 6 mM MgCl_2_, and 3 uL of the template DNA. An *R. solani* AG-1-IB assay was also conducted in a final volume of 25 uL containing 1X ECO master mix (Thermofisher), 400 nM each of the AG 1-IB-F3 and AG 1-IB-R primers, 200 nM of the AG 1-IB-P probe, 0.2 μg uL^−1^ of BSA, 6 mM MgCl_2_, 1% DMSO, and 3 uL of the template DNA. The qPCR cycling conditions for both qPCR assays were set at 95 °C for 5 min, followed by 40 cycles at 95 °C for 5 s and 62 °C for 20 s. The qPCR assays were performed using a QuantStudio 3 qPCR instrument (Thermofisher, Mississauga, ON, Canada), and each sample was run in duplicate on a 96-well plate containing a normalized standard curve. For both assays, the standard curves consisted of a 10-fold serial dilution ranging from 9 to 900,000 ITS copies. For the AG1-IB assay, a standard was built from a 150 bp double-stranded synthesized DNA gblock fragment from Integrated DNA Technologies (Coralville, IA, USA) based on the AG 1-IB ITS consensus sequence. For the *R. solani* assay, ITS1-ITS4-mixed PCR products of different AG strains (AG 1-IB, AG 2-2, AG 3, AG 4-HGII, AG 5, and AG 11) were used to capture as much variation as possible. Then, an exogenous internal control (IC) was multiplexed to each assay to detect PCR inhibition. The already published IC system EIPC100 was used as described in Reference [44]. The IC fragment consisted of double-stranded DNA genomic blocks designed from a random DNA sequence [44]. Three hundred nanomolar of each primer, 100 nM of the probe (Table 1), and a total of 10,000 copies of the gblock were added to each qPCR reaction. A comparison of the *R. solani* and AG-1-IB respective standard curves with or without IC was done to ensure that the duplex did not interfere with the assay sensitivity.

### 4.7. Specificity and Sensitivity of the Rhizoctonia solani and Rhizoctonia solani AG1-IB qPCR Assays

The specificity of the *R. solani* and *R. solani* AG1-IB assays was tested against the DNA of several *R. solani* samples belonging to different AG and Binucleate *Rhizoctonia* isolates collected in Québec from different host plants, as well as from ubiquitous fungi and other fungal organisms frequently isolated from lettuce (Table 2). A total of 0.1 ng of genomic DNA was used in each reaction. The sensitivity of both assays was determined using a 10-fold serial dilution ranging from 0.01 pg to 1000 pg of total *R. solani* DNA mix. The isolates U1097 (AG 1-IB), U512 (AG 2-2), U593 (AG 3), U578 (AG 4-HGII), U572 (AG-5), and P174 (AG 11) were used for the *R. solani* DNA mix, and isolates U117, U737, 333-J-1, 340-P8-1, 350-P7-1, 322-2-3, 302-1-2, 359-P1, and 352-P7-2 were used for the AG 1-IB DNA mix.

### 4.8. Validation with Artificially Inoculated Soil

The previously identified *Rhizoctonia solani* AG1-IB isolate 277.1 was grown on 90 mm Petri dishes containing PDA and incubated at room temperature for four weeks to promote sclerotia formation. At the end of the incubation period, the sclerotia were harvested, placed in a nylon mesh, washed under running water, and air-dried at room temperature for 48 h. The sclerotia were then sieved to keep only those with a diameter between 0.2 and 0.5 mm. Then, 300 mg of the sclerotia was thoroughly mixed to 30 g of dried organic soil to provide an initial concentration of 10,000 μg of sclerotia per gram of dry soil. From this concentration, a standard soil curve was prepared by mixing the inoculated soil with varying amounts of uninoculated soil to obtain concentrations ranging from 10,000 μg to 1 μg of sclerotia per gram of dry soil. A portion of the non-inoculated sterilized soil was used as a negative control. For each concentration, DNA extraction was performed as described in Section 4.3, and the DNA obtained was quantified using the qPCR assays developed in this project. This experiment was repeated four times.

### 4.9. Growth Chamber Assays

To verify that the qPCR detection limit in soil was not above the theoretical damage threshold, an inoculation experiment was performed under controlled conditions. The sclerotia were obtained from isolate 277.1 grown on PDA at room temperature for four weeks and harvested as described above. Inoculated soils were prepared to obtain concentrations of 0, 3, 8, 24, 72, 216, and 648 sclerotia in 800 g of fresh soil, corresponding to approximately 0, 5, 14, 42, 125, 375, and 1124 μg of sclerotia per g of dry soil. The inoculated soils were distributed in 1 L pots at the bottom, in which a 2 cm layer of sand was previously settled. A three-week-old lettuce plant (iceberg, c.v. Estival) was transplanted in each pot and then placed in a growth chamber following a randomized complete block design with four blocks and one replicate per concentration per block. Conditions in the growth chamber were set to 25 °C, with 80% relative humidity and a 14 h photoperiod. The plants were grown for 20 days and watered equally each day. The experiment was repeated twice.

Basal rot severity was evaluated for each plant every five days on a zero to three scale based on Grosch et al. (2010). A total of four evaluations were done in each experiment. Disease severity was evaluated using a four-category index (0—no distinct symptoms; 1—symptoms only on the first lower leaves in direct contact with the soil and small brown to dark spots primarily on the undersides of the leaf midribs; 2—brown spots on the leaf midribs in the lower and next upper leaf layers and rotting midribs and leaf blades; and 3—severe disease symptoms on the upper leaf layers and the beginning of head rot to total head rot) [7]. The area under the disease progress curve (AUDPC) was calculated to provide a single value that includes all the evaluations. The AUDPC was calculated as follows:

$$AUDPC\;=\;\overset{N_{i}-1}{\underset{i}{\sum }}\frac{\left(y_{i}+y_{i+1}\right)}{2}\left(t_{i+1}-t_{i}\right)$$

where $y_{i}$ is the disease severity at time $t_{i}$.

### 4.10. Statistical Analysis

Linear regression analysis was performed to describe the relationship and best fit lines for *R. solani* and *R. solani* AG1-IB gDNA and gBlock-based standard curves (Cq values against the log of the concentration), with and without an internal control. qPCR efficiency was calculated using the following equation:

E = 10^−(1/slope)^ − 1

The sensitivity and limits of detection in artificially inoculated soils were determined using an analysis of variance (ANOVA) followed by an LSD multiple comparison test. To characterize the relationship between inoculum density and disease severity in the artificial inoculation assay, an analysis of variance (ANOVA) followed by an LSD multiple comparison test was also performed. All statistical analyses were conducted using functions implemented in R (version 3.6.3).

## Figures and Tables

**Table 1:** Isolates of *Rhizoctonia solani,* Binucleate *Rhizoctonia,* and other pathogens used to test the specificity of the *R. solani* and *R. solani* AG 1-IB qPCR assays.

Isolate	Host	Species ^a^	AG	Genbank Accession	qPCR Assays ^e^	
					*R. solani*	*R. solani AG 1-IB*
U737 ^b^	*Lactuca sativa*	*Rhizoctonia solani*	1-IB	MT177242	22.70	22.42
U1097 ^b^	*Lactuca sativa*	*R. solani*	1-IB	MT177243	23.86	23.79
302-1-2	*Lactuca sativa*	*R. solani*	1-IB	MT177230	23.73	23.97
333-B-1 ^b^	*Lactuca sativa*	*R. solani*	1-IB	MT177231	25.12	25.20
356-P3-2A ^b^	*Lactuca sativa*	*R. solani*	1-IB	MT177232	23.00	23.38
361-P4 ^b^	*Lactuca sativa*	*R. solani*	1-IB	MT177233	23.62	23.70
362-P5-1 ^b^	*Lactuca sativa*	*R. solani*	1-IB	MT177234	23.14	23.67
434-1 ^b^	*Lactuca sativa*	*R. solani*	1-IB	MT177235	23.56	23.71
437-6 ^b^	*Lactuca sativa*	*R. solani*	1-IB	MT177236	22.30	22.92
472-2 ^b^	*Lactuca sativa*	*R. solani*	1-IB	MT177237	21.99	21.64
473-3 ^b^	*Lactuca sativa*	*R. solani*	1-IB	MT177238	21.58	21.59
475-1 ^b^	*Lactuca sativa*	*R. solani*	1-IB	MT177239	21.79	21.72
569-3 ^b^	*Lactuca sativa*	*R. solani*	1-IB	MT177240	23.56	23.38
570-1 ^b^	*Lactuca sativa*	*R. solani*	1-IB	MT177241	22.35	22.48
506-1 ^b^	*Lactuca sativa*	*R. solani*	1-IC	MT177244	23.47	–
642-6 ^b^	*Spinacia oleracea*	*R. solani*	1-IC	MT177245	26.77	–
370-P3-A ^b^	*Lactuca sativa*	*R. solani*	BI	MT177259	24.92	–
523-1 ^b^	*Lactuca sativa*	*R. solani*	BI	MT177260	24.53	–
284-1 ^b^	*Raphanus sativus*	*R. solani*	2-1	MT177246	25.62	–
U782 ^b^	*Spinacia oleracea*	*R. solani*	2-2	MT177248	23.93	–
U512 ^b^	*Daucus carota*	*R. solani*	2-2	MT177247	24.22	–
U500 ^b^	*Daucus carota*	*R. solani*	2-2-IV	MT177250	23.02	–
682-1 ^b^	*Daucus carota*	*R. solani*	2-2-IV	MT177249	28.91	–
U593 ^b^	*Solanum tuberosum*	*R. solani*	3	MT177251	24.90	–
U658 ^b^	*Solanum tuberosum*	*R. solani*	3	MT177252	24.53	–
341-2 ^b^	*Raphanus sativus*	*R. solani*	4-HGI	MT177253	22.64	–
U578 ^b^	*Daucus carota*	*R. solani*	4-HGII	MT177254	30.45	–
U572 ^b^	*Daucus carota*	*R. solani*	5	MT177256	25.76	–
390.Rs17-10 ^d^	*Glycine max*	*R. solani*	5	MT177255	29.81	–
P174 ^b^	*Pisum sativum* L.	*R. solani*	11	MT177257	25.09	–
U133 ^b^	*Lactuca sativa*	Binucleate *Rhizoctonia*	A	MT177258	–	–
390.Rs17-7 ^d^	*Glycine max*	Binucleate *Rhizoctonia*	E	MT177261	25.69	–
534-6 ^d^	*Triticum L.*	Binucleate *Rhizoctonia*	E	MT177262	27.24	–
Rs.23251 ^c^	* Fragaria × ananassa*	Binucleate *Rhizoctonia*	G	MT177263	–	–
534-2 ^d^	*Triticum L.*	Binucleate *Rhizoctonia*	H	MT177264	–	–
390.Rs16-70 ^d^	*Glycine max*	Binucleate *Rhizoctonia*	I	MT177265	–	–
390.Rs16-71 ^d^	*Glycine max*	Binucleate *Rhizoctonia*	K	MT177266	–	–
343-5A-12 ^b^	*Apium graveolens* L.	*Alternaria* sp.		MT177217	–	–
381-B2 ^b^	*Daucus carota*	*Botrytis cinerea*		MT177218	–	–
LDCC01 ^c^	ND	*Colletotrichum coccodes*		MT177219	–	–
U301 ^b^	*Lactuca sativa*	*Fusarium equiseti*		MT177220	–	–
U1416 ^b^	*Daucus carota*	*F. graminearum*		MT177221	–	–
U197 ^b^	*Lactuca sativa*	*F. oxysporum*		MT177222	–	–
357-P12-4B ^b^	*Lactuca sativa*	*F. solani*		MT177223	–	–
U709 ^b^	*Lactuca sativa*	*Mortierella* sp.		MT177224	–	–
280-3 ^b^	* Lactuca sativa*	*Pythium sylvaticum*		MT177227	–	–
U199 ^b^	*Lactuca sativa*	*P. irregulare*		MT177225	–	–
U191 ^b^	* Spinacia oleracea*	*P. oopapillum*		MT177226	–	–
U415 ^b^	*Daucus carota*	*P. sulcatum*		MH023358	–	–
647-3 ^b^	*Lactuca sativa*	*P. tracheiphilum*		MT177228	–	–
278-4 ^b^	*Spinacia oleracea*	*P. ultimum*		MT177229	–	–
381-C1 ^b^	*Daucus carota*	*Sclerotinia sclerotiorum*		MT177267	–	–
U795 ^b^	*Glycine max*	*Trichoderma* sp.		MT177268	–	–
Vda-18481 ^c^	ND	*Verticillium dahliae*		MT177269	–	–

^a^ All species and AG group were determined by ITS (internal transcribed spacer) or EF (elongation factor alpha) sequencing. ^b^ Isolate from Phytodata collection. ^c^ Isolate provided by the Laboratoire d’expertise et de diagnostic en phytoprotection (MAPAQ). ^d^ Isolate provided by the Centre de recherche sur les grains (CEROM). ^e^ Negative reactions are indicated as –, and unexpected amplifications are in bold.

**Table 2:** Primers and probes used in this study.

Target	Primers and Probes	5′-3′ Nucleotide Sequence	Reference
ITS	ITS1	TCC GTA GGT GAA CCT GCG G	[30]
	ITS4	TCC TCC GCT TAT TGA TAT GC	
EF	EF1	ATG GGT AAG GAR GAC AAG AC	[31]
	EF2	GGA RGT ACC AGT SAT CAT GTT	
	GMRS3-R	AGT GTT ATG CTT GGT TCC ACT	
*R. solani*	GRSM4_M_	CGG TTC RTC TGC ATT TAC CTT	Modified from [32]
	GRMP	*FAM*-CRG CGT GAT AAR TTA TCT ATC GC	This study
	AG 1-IB-F3	TGG CCT TTT AAC ATT GGC ATG T	This study
*R. solani* AG 1-IB	AG 1-IB-R	CCA ACC CCA AAG GAC CTT GA	
	AG 1-IB-P	*VIC*-CAC ACA CCC CTG TGC ACT TGT GAG AC	
	EIPC1mt100F	AGG CTA GCT AGG ACC GAT CAA TAGG	[33]
IC-EIPC100	EIPC1mt100R	AGT GCT TCG TTA CGA AAG TGA CCT TA	
	EIPC1mt100P	*ABY*-CCT ATG CGT TCC GAG GTG ACG ACC TTG CC	

**Table 3:** Validation of the detection of *Rhizoctonia solani* and *Rhizoctonia solani* AG1-IB in field lettuce samples presenting bottom rot symptoms by molecular assay with qPCR for which *R. solani* isolates were identified by culture method.

Grower	Field	Sample	AG ^a^ Isolated	Lettuce Type ^b^	qPCR Assays ^c^	
					*R. solani*	*R. solani* AG 1-IB
1	A	437	1-IB	R	+	+
		439	1-IB	R	+	+
		472	1-IB	R	+	+
2	B	438	1-IB	R	+	+
		469	1-IB	R	+	+
	C	434	1-IB	I	+	+
	D	564	1-IB	I	+	+
		566	1-IB	I	+	+
		567	1-IB	I	+	+
		568	1-IB	I	+	+
		569	1-IB	I	+	+
		570	1-IB	I	+	+
		571	1-IB	I	+	+
		370-P4	1-IB	R	+	+
		370-P3	BI	R	+	−
	E	523	BI	R	+	−
	F	369-P2	1-IB	R	+	+
		369-P7	1-IB	R	+	+
3	G	362-P5-1	1-IB	I	+	+
	H	337-P2	1-IB	I	+	+
	I	506	1-IC	I	+	−
	J	551	1-IB	I	+	+
		552	1-IB	I	+	+
4	K	333-B	1-IB	ND	+	+
		333-E	1-IB	ND	+	+
		333-J	1-IB	ND	+	+

^a^ AG group of the isolates determined by ITS sequencing. ^b^ R: Romaine, I: Iceberg lettuce cultivars, ND: not determined. ^c^ Negative reactions are indicated as −.

## Data Availability

The data presented in this study are available as supplementary material.

## Supplementary Materials

The following are available online at https://www.mdpi.com/2223-7747/10/1/57/s1, Figure S1: Sequence alignment of the internal transcribed spacer 1 region for *Rhizoctonia solani* strains collected from lettuce in this study. Figure S2: Sequence alignment of *Rhizoctonia solani* internal transcribed spacer sequences for AG1-IB and AG-BI. Supplementary Figure S3: Diagram showing the position of the AG1-IB and *R. solani* assay on the internal transcribed spacer region. Figure S4: Sequence alignment of *Rhizoctonia solani* internal transcribed spacer sequences used for the design of the GRMP probe in combination with the existing GRSM3-GRSM4 primers. Figure S5: Sequence alignment of internal transcribed spacer sequences for the AG1 sub-group. Figure S6: Alignment of *R. solani* internal transcribed spacer sequences used for the design of the AG1-IB primers and probe. Figure S7: Examples of PCR product visualisation on 2% agarose gel for the AG 1-IB qPCR assay with DNA from various AGs and other lettuce pathogens. Figure S8: Examples of PCR product visualisation on 2% agarose gel for the *R. solani* qPCR assay with DNA from various AGs and other lettuce pathogens. Table S1: Cycle quantification threshold (Cq) values obtained for the gDNA standard curves presented in Figure 2A for the AG1-IB assay. Table S2: Cycle quantification threshold (Cq) values obtained for the gDNA standard curves presented in Figure 2B for the *R. solani* assay. Table S3: Cycle quantification threshold (Cq) values obtained for the amplicon standard curves presented in Figure 3 for the AG1-IB and the *R. solani* assay. Table S4: Cycle quantification threshold (Cq) values obtained for the amplicon standard curves with and without internal control, as presented in Figure 3 for the AG1-IB and the *R. solani* assay. Table S5: Cycle quantification threshold (Cq) values obtained for the standard curves obtain from artificial inoculation of sterilized soil, as presented in Figure 4 for the AG1-IB and the *R. solani* assay.
