# Supplementary material for: Detection and Quantification of Rhizoctonia solani and Rhizoctonia solani AG1-IB Causing the Bottom Rot of Lettuce in Tissues and Soils by Multiplex qPCR
Source: Plants (Basel). 2020 Dec 29;10(1):57. doi: 10.3390/plants10010057 (PMC7824208; doi:10.3390/plants10010057)
Supplement: Supplementary file 1 [file plants-10-00057-s001.pdf]

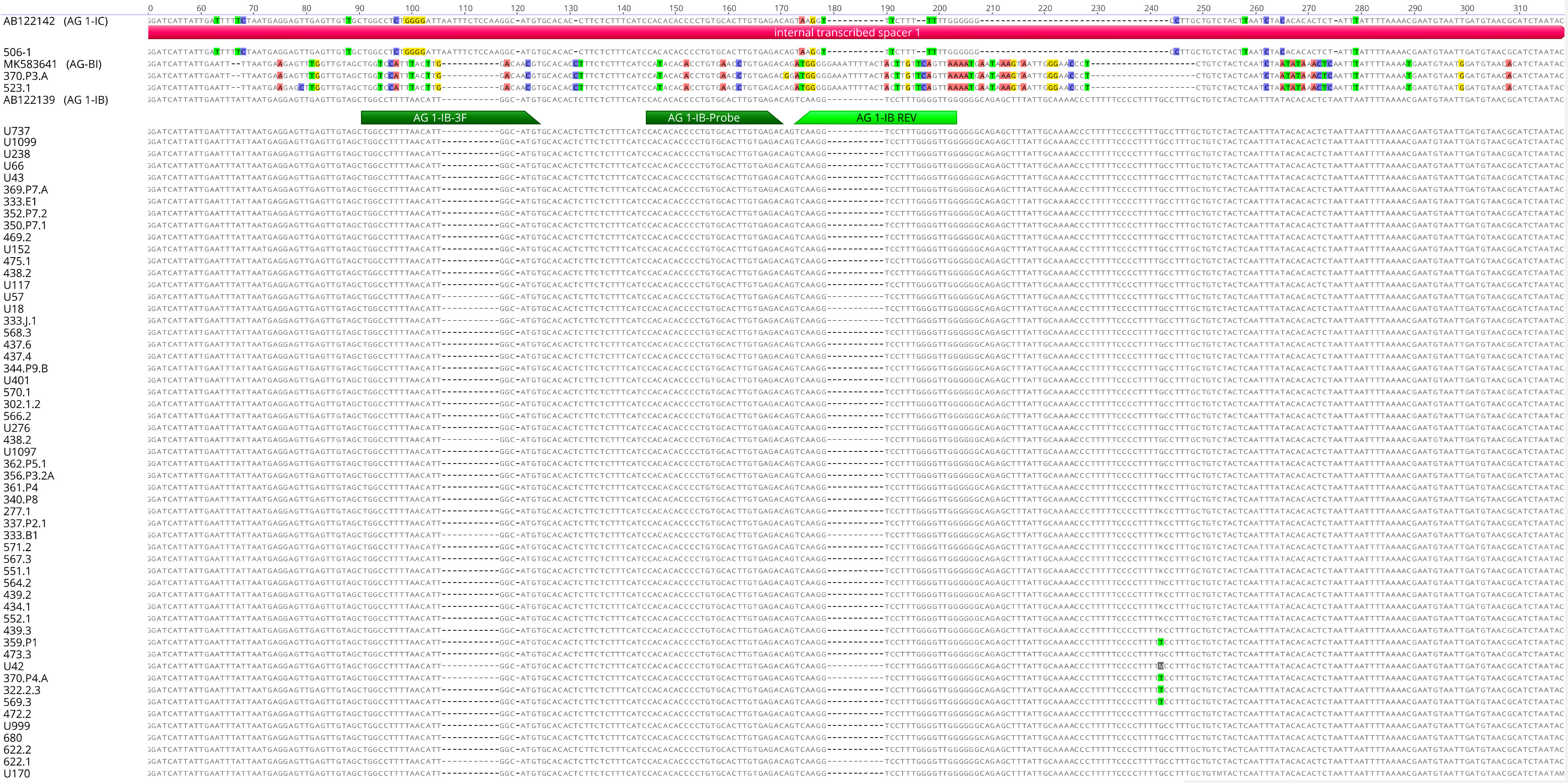
AB122142 (AG 1-IC)
internal transcribed spacer 1
506-1
MK583641 (AG-BI)
370.P3.A
523.1
AB122139 (AG 1-IB)
AG 1-IB-3F
AG 1-IB-Probe
AG 1-IB REV
U737
U1099
U238
U66
U43
369.P7.A
333.E1
352.P7.2
350.P7.1
469.2
U152
475.1
438.2
U117
U57
U18
333.J.1
568.3
437.6
437.4
344.P9.B
U401
570.1
302.1.2
566.2
U276
438.2
U1097
362.P5.1
356.P3.2A
361.P4
340.P8
277.1
337.P2.1
333.B1
571.2
567.3
551.1
564.2
439.2
434.1
552.1
439.3
359.P1
473.3
U42
370.P4.A
322.2.3
569.3
472.2
U999
680
622.2
622.1
U170

Supplementary Figure S1. Sequence alignment of the internal transcribed spacer 1 region for *Rhizoctonia solani* strains collected from lettuce in this study.

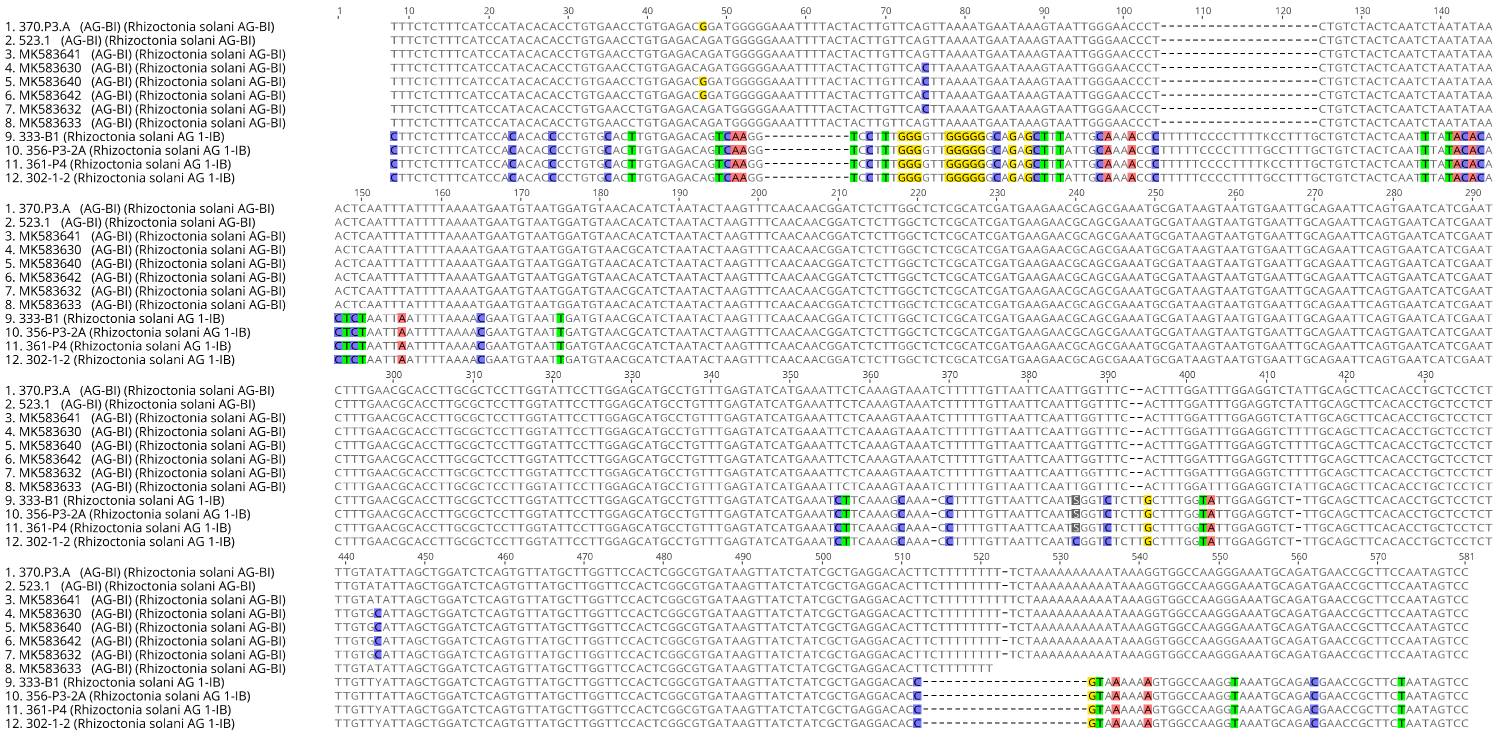

Supplementary Figure S2. Sequence alignment of *Rhizoctonia solani* internal transcribed spacer sequences for AG1-IB and AG-BI.

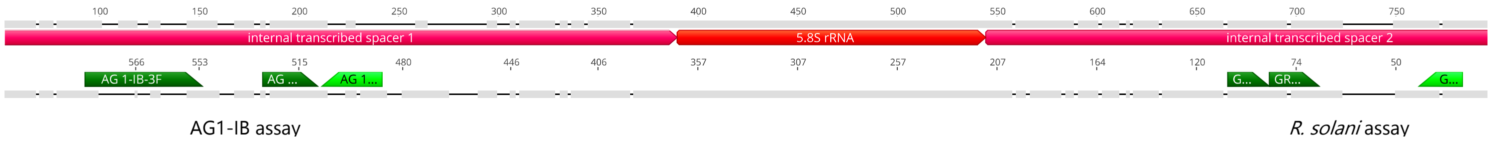

Supplementary Figure S3. Diagram showing the position of the AG1-IB and *R. solani* assay on the internal transcribed spacer region.

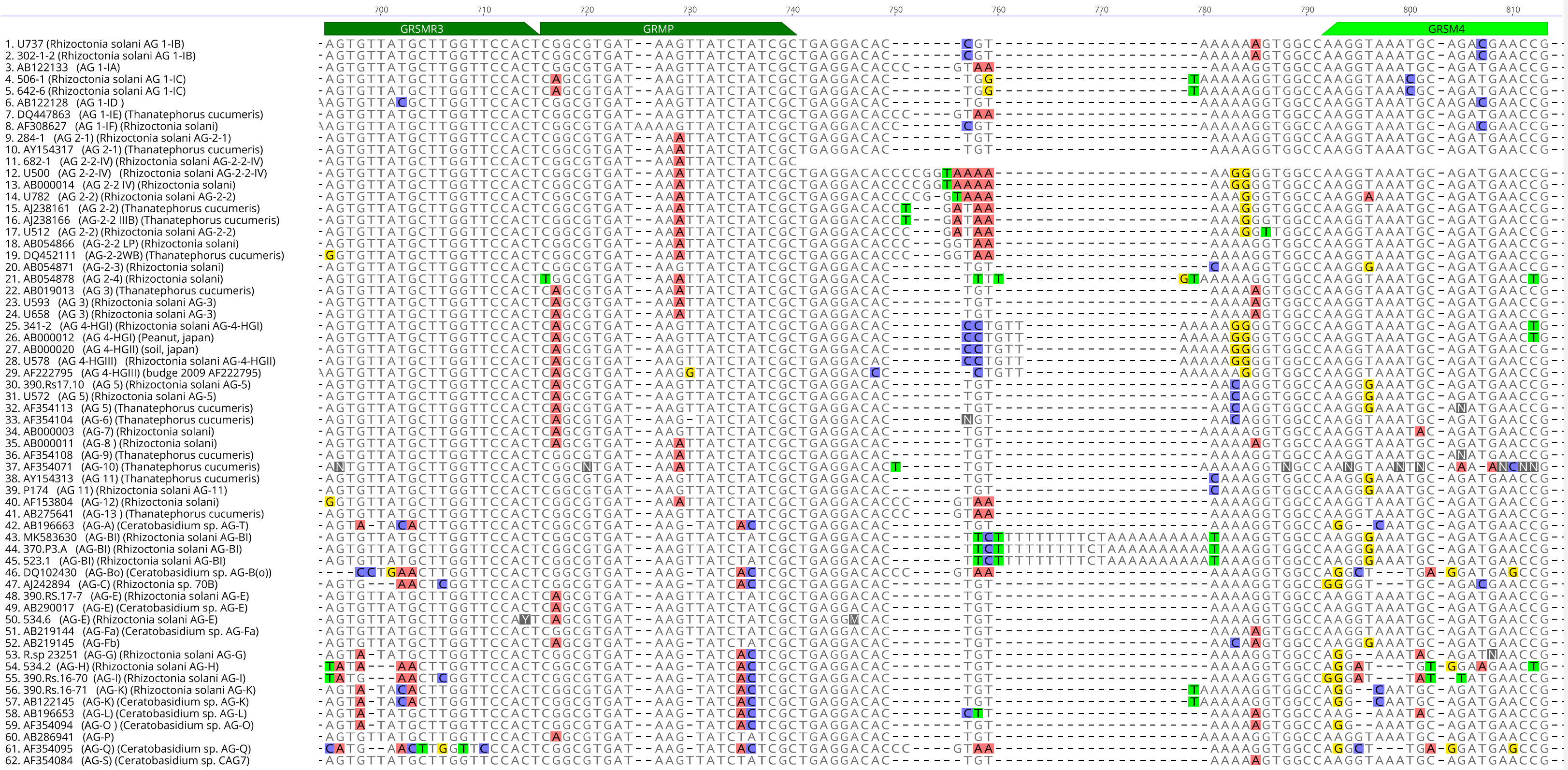

Supplementary Figure S4. Sequence alignment of *Rhizoctonia solani* internal transcribed spacer sequences used for the design of the GRMP probe in combination with the existing GRSM3-GRSM4 primers.

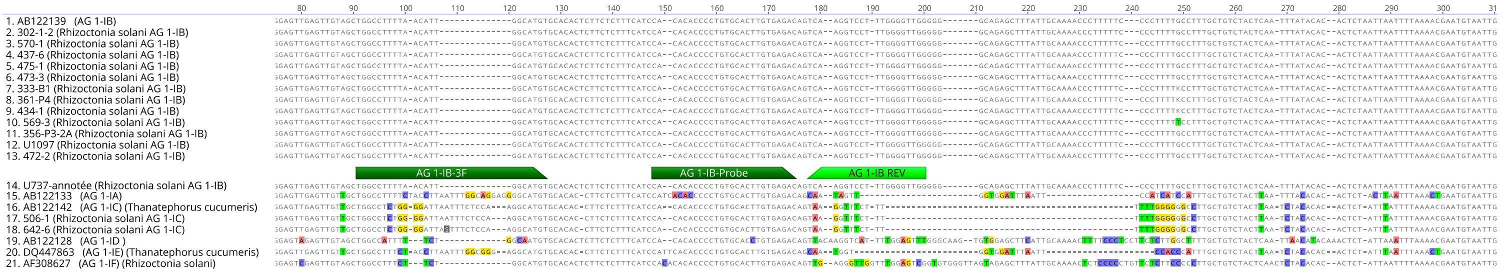

Supplementary Figure S5. Sequence alignment of internal transcribed spacer sequences for the AG1 sub-group.

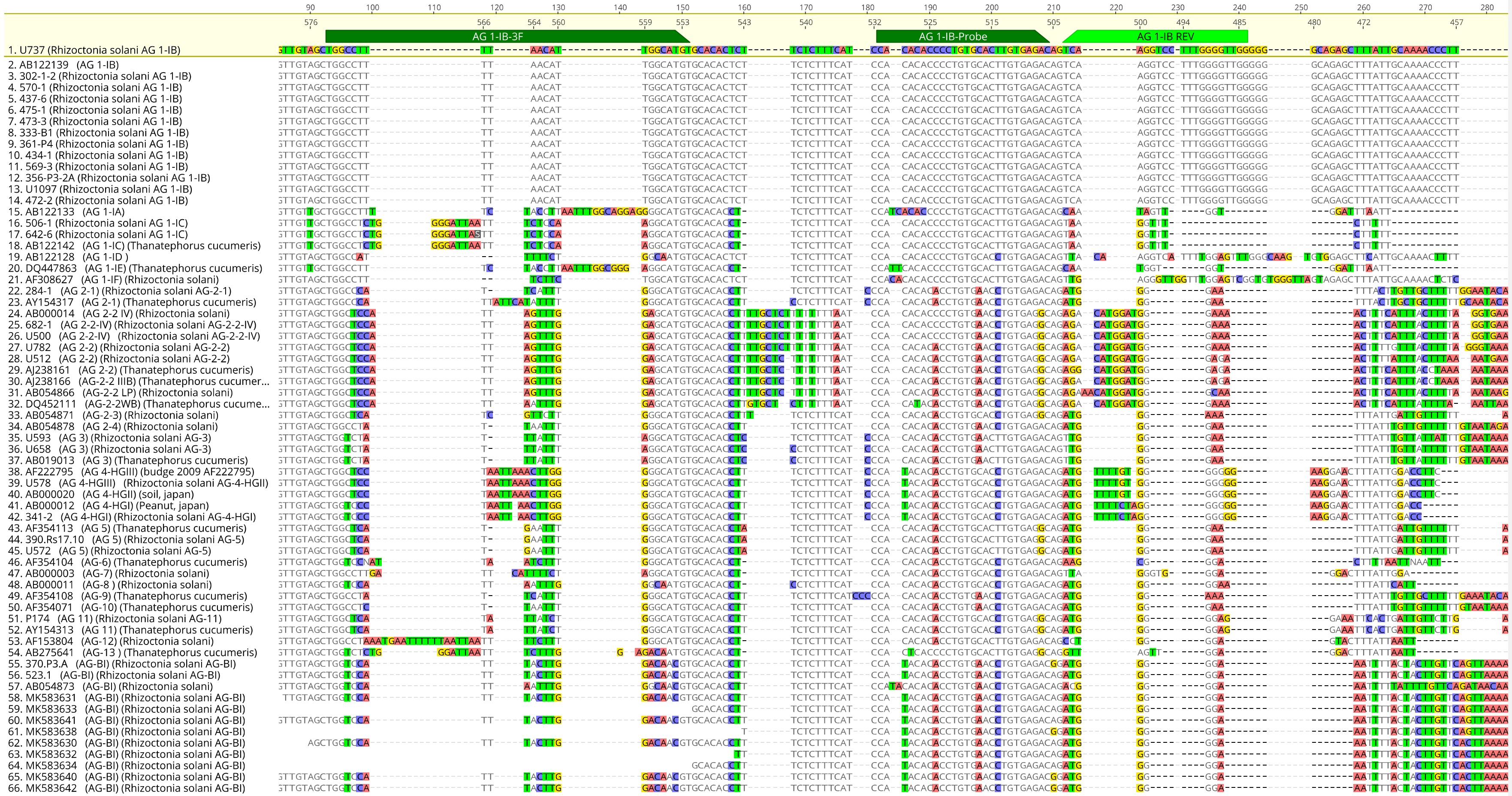

Supplementary Figure S6. Alignment of *R. solani* internal transcribed spacer sequences used for the design of the AG1-IB primers and probe

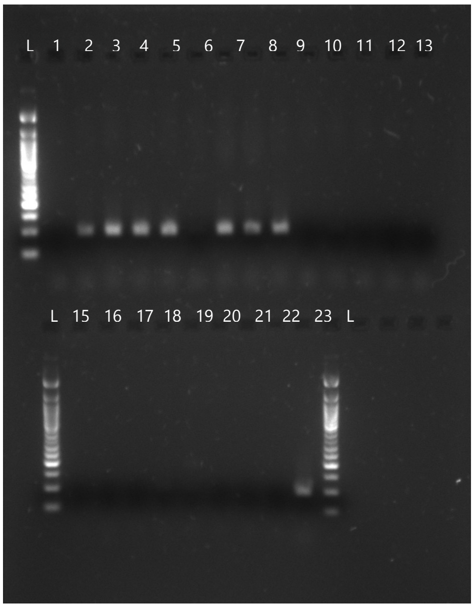

Supplementary Figure S7. Examples of PCR product visualisation on 2% agarose gel for the AG 1-IB qPCR assay with DNA from various AGs and other lettuce pathogens. Lanes L: 50pb DNA ladder; 1: $H_2$0; 2: 900 copies of Ag-1-IB; 3: 333-J-1 (AG1-IB); 4: 340-P8.1(AG 1-IB); 5: 350.P7.1 (RsAG 1-IB); 6: 370.P3.A (AG BI); 7: U737 (AG 1-IB); 8: U117 (AG 1-IB); 9: U1097(AG 1IB); 10: U500 (AG –2-2-IV); 11: U572 (AG 5); 12: U578 (AG 4 HGII4); 13: U658 (AG-3); 14: P174 (AG-11); 15: U785 (AG A); 16: Rs.23251 (AG-G); 17: 280-3 (*Pythium sylvaticum*); 18: 316-4 (*P. tracheiphilum*); 19: U709 (*Mortierella sp.*); 20: U301 (*Fusarium equiseti*); 21: 343-5A.-2 (*Alternaria sp.*); 22: U795 (*Trichoderma sp.*); 23: 335-1 (AG-1-IB).

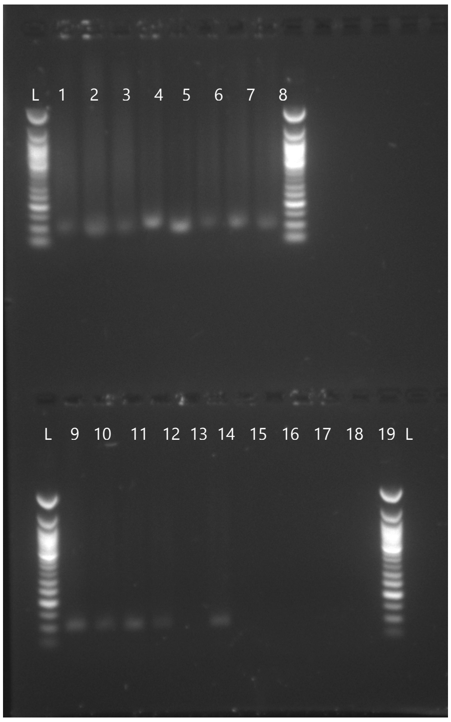

Supplementary Figure S8. Examples of PCR product visualisation on 2% agarose gel for the *R. solani* qPCR assay with DNA from various AGs and other lettuce pathogens. Lanes L: 50pb DNA ladder; 1: U1097 (AG1-IB); 2: 333-B-1 (AG1-IB); 3: 506.1 (AG1-IC); 4: 370-P3-A (AG BI); 5: U650 (AG2-1); 6: U782 (AG2-2); 7: U682 (AG2-2-IV); 8: U593 (AG3); 9: 341-2 (AG4-HGI); 10: U578 (AG4-GII4); 11: U572 (AG 5); 12: P1263 (AG11); 13: 567 (AG-A); 14: 534-6 (AG-E); 15: 534-5 (AG G); 16: 534-2 (AG-H); 17: 390RS16.70 (AG-I); 18: 390RS16.71 (*AG-K*); 19: $H_2$0.

Supplementary Table S1. Cycle quantification threshold (Cq) values obtained for the gDNA standard curves presented in Figure 2A for the AG1-IB assay.

	Experiment 1		Experiment 2		Experiment 3	
gDNA (pg)	Technical Rep. 1	Technical Rep. 2	Technical Rep. 1	Technical Rep. 2	Technical Rep. 1	Technical Rep. 2
1000	19.391	19.612	19.569	19.640	19.451	19.834
100	23.267	23.068	23.095	23.094	23.267	23.278
10	26.849	26.549	26.489	26.662	26.923	26.856
1	29.835	29.879	30.180	30.067	30.193	30.167
0	32.851	33.535	33.250	33.303	33.474	32.956

Commented [M1]: There is no mention of supplementary tables in the main text.

Commented [hvdh2R1]: Tables are cited now

Supplementary Table S2. Cycle quantification threshold (Cq) values obtained for the gDNA standard curves presented in Figure 2B for the *R. solani* assay.

gDNA (pg)	AG5 U572		AG2-2 U512		AG1-IB U1097		AG11 P174		AG4 HGII U578		AG3 U593	
	Technical Rep. 1	Technical Rep. 2	Technical Rep. 1	Technical Rep. 2	Technical Rep. 1	Technical Rep. 2	Technical Rep. 1	Technical Rep. 2	Technical Rep. 1	Technical Rep. 2	Technical Rep. 1	Technical Rep. 2
1000	17.89	17.97	17.00	16.87	19.64	19.92	19.33	19.05	19.16	18.77	16.53	16.56
100	21.19	21.12	20.26	20.26	22.82	22.51	22.08	22.08	21.96	22.25	19.74	19.34
10	24.55	24.54	23.53	23.53	26.12	26.05	25.40	25.13	25.52	25.65	23.20	22.83
1	27.85	27.77	26.90	26.80	29.78	29.65	28.61	28.57	29.13	28.80	26.73	26.32
0.10	31.09	31.05	30.47	30.26	33.00	32.51	31.94	31.91	32.32	32.23	30.22	29.63

Supplementary Table S3. Cycle quantification threshold (Cq) values obtained for the amplicon standard curves presented in Figure 3 for the AG1-IB and the *R. solani* assay.

		Experiment 1		Experiment 2		Experiment 3	
ITS copy number	Target	Technical Rep. 1	Technical Rep. 2	Technical Rep. 1	Technical Rep. 2	Technical Rep. 1	Technical Rep. 2
900000	AG1-IB	19.26	19.16	19.36	19.23	19.62	19.23
90000	AG1-IB	22.59	22.61	22.63	22.63	22.63	22.83
9000	AG1-IB	25.95	26.03	26.00	26.06	26.01	26.10
900	AG1-IB	29.47	29.30	29.47	29.20	29.34	29.34
90	AG1-IB	32.41	32.51	32.55	32.79	32.55	33.17
9	AG1-IB	35.66	37.07	36.98	34.95	36.52	36.15
900000	*R. solani*	18.41	18.39	17.47	17.66	17.52	17.36
90000	*R. solani*	21.60	21.52	20.76	20.92	20.80	20.84
9000	*R. solani*	24.99	25.07	24.16	24.20	24.20	24.23
900	*R. solani*	28.37	28.25	27.57	27.55	27.56	27.53
90	*R. solani*	31.90	ND	30.95	30.87	31.20	30.82
9	*R. solani*	35.10	34.72	34.08	34.33	33.96	33.74

Supplementary Table S4. Cycle quantification threshold (Cq) values obtained for the amplicon standard curves with and without internal control, as presented in Figure 3 for the AG1-IB and the *R. solani* assay.

ITS copy number	Target	Experiment 1		Experiment 2		Experiment 3	
		Technical Rep. 1	Technical Rep. 2	Technical Rep. 1	Technical Rep. 2	Technical Rep. 1	Technical Rep. 2
900000	*R. solani*	18.56	18.46	17.51	17.53	17.42	17.58
90000	*R. solani*	21.70	21.73	20.93	20.86	20.77	20.89
9000	*R. solani*	25.16	25.10	24.11	24.19	24.17	24.17
900	*R. solani*	28.17	28.19	27.45	27.43	27.55	27.53
90	*R. solani*	31.65	31.30	31.28	30.65	30.72	30.81
9	*R. solani*	35.12	34.21	35.07	33.97	33.30	33.18
900000	IC	26.28	26.32	26.54	26.46	26.41	26.37
90000	IC	26.23	26.26	26.50	26.59	26.37	26.46
9000	IC	26.36	26.29	26.55	26.41	26.48	26.42
900	IC	26.31	26.28	26.50	26.58	26.51	26.49
90	IC	26.46	26.34	26.44	26.47	26.54	26.49
9	IC	26.50	26.34	26.51	26.51	26.31	26.50
900000	AG1-IB	19.04	19.35	19.06	19.23	18.97	19.00
90000	AG1-IB	22.62	22.73	22.74	22.82	22.76	22.75
9000	AG1-IB	26.13	26.10	26.06	26.12	26.16	26.25
900	AG1-IB	29.43	29.34	29.45	29.37	29.51	29.53
90	AG1-IB	32.49	32.80	33.16	33.08	32.98	33.17
9	AG1-IB	38.55	37.31	36.65	36.18	37.14	36.90
900000	IC	26.33	26.46	26.32	26.46	26.49	26.50
90000	IC	26.33	26.25	26.50	26.36	26.49	26.40
9000	IC	26.21	26.22	26.32	26.11	26.25	26.30
900	IC	26.15	26.09	26.12	26.11	26.24	26.14
90	IC	26.09	25.98	26.11	26.13	26.30	26.29
9	IC	26.08	26.07	26.16	26.15	26.25	26.27

Supplementary Table S5. Cycle quantification threshold (Cq) values obtained for the standard curves obtain from artificial inoculation of sterilized soil, as presented in Figure 4 for the AG1-IB and the *R. solani* assay.

		Experiment 1		Experiment 2		Experiment 3		Experiment 4	
ug sclerotia g^{-1} soil	Target	Technical Rep. 1	Technical Rep. 2	Technical Rep. 1	Technical Rep. 2	Technical Rep. 1	Technical Rep. 2	Technical Rep. 1	Technical Rep. 2
10000	AG1-IB	15.99	16.65	16.88	16.96	18.05	18.11	18.39	18.73
1000	AG1-IB	24.34	24.40	22.14	22.24	20.80	20.81	20.72	20.71
100	AG1-IB	30.42	30.68	24.30	24.72	28.14	28.23	28.42	28.82
10	AG1-IB	29.88	30.04	30.76	30.42	34.13	34.35	32.11	33.07
1	AG1-IB	35.64	ND	34.63	34.40	33.53	33.46	35.64	34.95
10000	IC	25.41	25.51	25.51	25.58	25.78	25.91	25.97	25.86
1000	IC	25.79	25.58	25.65	25.65	25.83	25.67	25.91	25.75
100	IC	25.54	25.56	25.58	25.64	25.79	25.82	25.82	25.79
10	IC	25.62	25.60	25.72	25.54	25.97	25.77	25.80	25.86
1	IC	25.62	25.44	25.61	25.58	25.73	25.84	25.84	25.87
10000	*R. solani*	18.59	18.92	18.92	19.21	19.99	20.29	20.39	19.92
1000	*R. solani*	26.40	26.43	24.20	24.39	22.94	22.91	22.84	22.92
100	*R. solani*	32.21	32.58	26.46	26.88	29.96	30.30	30.28	30.63
10	*R. solani*	32.06	32.07	31.77	31.61	33.94	34.47	33.37	34.04
1	*R. solani*	ND	36.90	34.82	35.22	34.03	35.33	33.98	34.03
10000	IC	25.26	25.29	24.74	24.96	25.13	25.24	25.14	25.02
1000	IC	25.49	25.15	25.07	25.10	25.31	25.13	25.21	25.15
100	IC	25.26	25.19	25.10	25.13	25.25	25.27	25.48	25.38
10	IC	25.36	25.23	25.19	25.23	25.27	25.24	25.25	25.25
1	IC	25.30	25.15	25.15	25.21	25.17	25.01	25.15	25.23
